# Editorial: Metabolism and Cell Adhesion in Cancer

**DOI:** 10.3389/fcell.2022.871471

**Published:** 2022-03-03

**Authors:** Tanja Nicole Hartmann

**Affiliations:** Department of Internal Medicine I, Faculty of Medicine and Medical Center, University of Freiburg, Freiburg, Germany

**Keywords:** tumor microenvironment, adhesion, metabolism, integrin, migration, AMPK, mTOR, FAK

Cell adhesion is a critical component of malignant transformation, cancer progression, and the development of chemoresistance. Adhesion molecules allow tumor cells to invade the tissues surrounding the primary tumor and to extravasate during metastasis. In addition, adhesion molecules serve as anchors that position tumor cells in close proximity to immune and stromal cells. This positioning is required to establish intercellular connections and communication, which eventually re-educates the tumor microenvironment to support the cancer.

Adhesion and mechanotransduction are closely linked. Adhesion molecules enable cells to sense the physical properties of the extracellular matrix and integrate mechanical stimuli into cellular functions. This intracellular signal transduction downstream of adhesive contacts is energy-dependent. During cancer progression, malignant cells are surrounded by increasing levels of tissue pressure, density, and hypoxia, as well as altered ATP levels, reflecting the increased nutrient demand that is characteristic of malignant cells.

Emerging evidence suggests that cell adhesion could be operating under the control of cell metabolism. Indeed, the second messengers and signaling pathways that control cellular respiration and nucleotide synthesis are similar to those controlling immune and cancer cell adhesion. However, we are just beginning to understand some of the common molecular links connecting metabolism and adhesion in cancer, which is underlined by the articles in this Research Topic.

Urra et al. describe in their review article factors arising from the remodeling of the extracellular matrix of the tumor, which is characterized by modulations of the stiff tumor microenvironment. They describe how these modulations are linked to the malignant phenotype regarding mitochondrial oxidative phosphorylation, glutamine consumption, and increased production of mitochondrial reactive oxygen species. Among the key molecular hubs linking the migratory and metabolic transduction are focal adhesion kinase (FAK), 5′ adenosine monophosphate-activated protein kinase (AMPK), a highly conserved sensor of intracellular adenosine nucleotide levels, and its downstream target mammalian target of rapamycin (mTOR). These molecules are also central components of the bone marrow microenvironment of acute leukemias, where the interplay between cell migration, adhesion, and metabolic components is indispensable for bone marrow niche stability, as described by Ashok et al., AMPK is thereby a major link between integrins and metabolism, while serving as a stress sensor.

In acute lymphoblastic leukemia, central nervous system (CNS) involvement is a major clinical concern. Some of the major adhesive players of the bone marrow, e.g., the integrin very late antigen-4 and various other integrins and the downstream components described above, play relevant roles in the CNS microenvironment. However, the metabolic adaptations to create the permissive microenvironment for leukemia adhesion and thus colonization in this nutrition-poor environment are not yet understood well, as outlined by Sharma et al.

Garcia et al. focus on the brain microenvironment and its metabolic changes. The authors discuss the signaling pathways involved in glioblastoma metabolism and their contribution to tumor cell invasion. One example is the link between glutamine-related pathways and mTOR complex 1 (mTORC1) signaling. The authors illustrate the recent dynamic view of the complexity of interactions of the genotype and the microenvironment.

In their original report, Joechle et al. provide evidence that dual pharmacological inhibition of mTORC1 and mTORC2 has the potential to diminish oncogenic signaling in intrahepatic cholangiocarcinoma cells. Matrix metalloproteinases (MMPs) are involved in the degradation of the tumor environment to release tumor cells for migration. In this work, the authors establish a link of tumor cell motility within the extracellular matrix, modulation of MMP2 and MMP9 expression and mTORC inhibition. Dual inhibition of mTORC might offer a useful option to circumvent the compensatory upregulation of survival signaling and accompanied resistance to current therapy strategies.

Previous data show a link between mTOR and autophagy. Upon nutrient starvation and stress condition, mTOR is itself inhibited, which results in induction of autophagy. Emerging evidence suggests an involvement of autophagy in cancer cell motility and invasiveness and in the modulation of dynamic cell adhesion, particularly in the context of epithelial-to-mesenchymal transition. Garcia-Miranda et al. dissect in their original report how the adipokine leptin influences tumor cell migration of breast cancer cell lines and suggest a role for basal autophagy or leptin-induced autophagy in leptin-induced migration and ERK phosphorylation, linking obesity and cancer cell migration.

Lamins are multifunctional proteins that are often aberrantly expressed or localized in tumors and bridge mechanical forces to the cell nucleus. Misregulation or mutations in lamin, particularly lamin A can be connected to the disregulation of AMPK/TOR/autophagy signaling. The nature of lamin function in cancer is, however, very complex and its role in tumor cell migration is controversially discussed. Urciuoli et al. contribute to this discussion by providing evidence that modulating Lamin A/C expression impacts on osteosarcoma cell aggressiveness and adhesion properties. They suggest considering the mechano-environment of osteosarcoma as a crucial factor in the onset, development, and progression of this bone tumor.

In summary, in this research topic we have presented a collection of articles focusing on how adhesion and metabolism, two facets of the tumor microenvironment, influence each other. By describing the currently known key components of this interplay, the authors hope to contribute to a deeper understanding and better characterization of this important but not yet well understood field.

